# Editorial: Experimental and Clinical Approaches in the Pursuit of Novel Therapeutic Strategies for Perinatal Brain Injury and Its Neurological Sequelae

**DOI:** 10.3389/fncel.2021.762111

**Published:** 2021-10-11

**Authors:** Changlian Zhu, Chao Chen, Claire Thornton

**Affiliations:** ^1^Henan Key Laboratory of Child Brain Injury and Henan Pediatric Clinical Research Center, Institute of Neuroscience and Third Affiliated Hospital of Zhengzhou University, Zhengzhou, China; ^2^Center for Brain Repair and Rehabilitation, Institute of Neuroscience and Physiology, Sahlgrenska Academy, University of Gothenburg, Gothenburg, Sweden; ^3^Department of Neonatology, Children's Hospital of Fudan University, Shanghai, China; ^4^Department of Comparative Biomedical Sciences, Royal Veterinary College, London, United Kingdom

**Keywords:** perinatal brain injury, white matter injury, neuroprotection, cerebral palsy, autism, intellectual disability, cell death, preterm birth

Brain injuries during the perinatal period—resulting from encephalopathy-related asphyxia, prematurity, fetal growth restriction, antenatal toxicity exposure, neonatal stroke, systemic infections, and metabolic abnormalities—are a significant cause of adverse neurodevelopmental outcomes, and such injuries occur at different times during the perinatal period and in different clinical settings (Leaw et al., 2017). Perinatal brain injury causes developmental impairment and permanent neurological deficits such as cerebral palsy, autism, and intellectual disability in children, and these are major burdens to those afflicted, their families, and society as a whole. The progression of perinatal brain injury depends on the balance between persistent injury and the repair response, which can be modulated by external therapy (Nair et al., 2021; Song et al., 2021). Interventions against brain injury using hypothermia or recombinant human erythropoietin during the neonatal period have shown promising results in reducing the prevalence of cerebral palsy and other neurological sequelae such as autism, intellectual disability, and retinopathy of prematurity (Shankaran et al., 2012; Song et al., 2016). However, these interventions are not successful in all cases, especially in very preterm infants, and the current therapy for preterm brain injury is still mainly supportive (Juul et al., 2020). Therefore, there is a pressing need for a better understanding of the mechanisms of perinatal brain injury in order to develop strategies for conducting comparative and translational studies on how to reduce brain injuries and how to promote injury repair and improve neurological outcomes in both term and preterm infants.

This Research Topic is dedicated to understanding the influence of perinatal brain injuries on neurological complications, specifically the insult-induced cell death and inflammation signaling pathways. From this wider understanding, novel experimental intervention strategies could be hypothesized and developed. This Research Topic comprises 16 articles representing the work of 131 authors, including 7 animal studies, 3 clinical studies, 5 reviews, and 1 meta-analysis. Of the 7 animal studies, the majority used rodents, and 1 study used fetal sheep. Hypoxic-ischemic brain injury was the most frequently used experimental model (3 articles) in this Research Topic collection. Other models included fetal growth restriction (FGR) (2 articles), germinal matrix hemorrhage (1 article), and perinatal toxicity exposure (1 article).

Hypoxic ischemic encephalopathy is a subtype of perinatal brain injury and is one of the major contributors to global neonatal morbidity and mortality. Brain injury resulting from birth asphyxia-related hypoxia-ischemia (HI) is associated with the development of cerebral palsy and cognitive deficits in those who survive such injuries (Zhang et al., 2020). HI induces neuronal cell death (Thornton et al., 2017) that persists for a long period and is related to inflammation and epigenetic dysregulation (Ma and Zhang, 2015; Albertsson et al., 2018). Recently, drugs used for the treatment of type 2 diabetes were found to have neuroprotective properties and therapeutic efficacy against neurological sequelae in an HI model, thus showing promising clinical translation potential (Rocha-Ferreira et al., 2018). Poupon-Bejuit et al. summarize recent findings on the use of diabetes drugs for the treatment of neonatal HI brain injury and indicate that diabetes drugs might be considered for clinical translation as a potential treatment. Epigenetic regulation plays an essential role during development, and perinatal insult-induced brain injury and subsequent pathological processes have been associated with lasting disruptions to the epigenetic control of gene expression contributing to neurological dysfunction. Bustelo et al. reviewed the current understanding of epigenetic mechanisms in perinatal HI brain injury and the potential clinical implications in terms of prognostic evaluation and therapeutic interventions. However, knowledge on the role of epigenetics in HI is still very limited and more efforts are needed for continued explorations in this area. Inflammation has been suggested to be a major etiological factor in perinatal brain injury (Hagberg et al., 2015), and innate lymphoid cells have been shown to play an important role in neuroinflammation (Nazmi et al., 2018). Zelco et al. investigated whether type 2 innate lymphoid cells contribute to the development of preterm brain injury in a mouse HI model. They found that even though innate lymphoid cells accumulate in the injured brain hemisphere, type 2 innate lymphoid cells do not contribute to the development of brain injury in the mouse model of preterm brain injury. High-mobility group box 1 (HMGB1) promotes neurite outgrowth and thus promotes brain development under physiological conditions. However, under pathological conditions, HMGB1 can act as a pro-inflammatory factor and can promote brain damage. Sun et al. studied the role of HMGB1 in neonatal rat HI-induced brain injury and found that it can lead to an imbalance in microglial polarization, suggesting that HMGB1 might be used as a therapeutic target against neonatal HI brain injury. To test whether perinatal HI results in deficits in complex cognitive and executive function, Maxwell et al. used a touchscreen platform to assess cognitive function in mice at postnatal day (P)70 (adulthood) following neonatal HI administered at P10 and showed that HI insult in the neonatal period is sufficient to cause long-lasting impairments in cognition and learning processes.

Preterm intraventricular hemorrhage (IVH) occurs in nearly half of infants born at <26 weeks' gestation, and 50–75% of survivors of IVH develop neurological disabilities. Moreover, around 25% of non-disabled survivors develop psychiatric disorders and problems with executive function (Luu et al., 2009; Stoll et al., 2015). Currently, no widely accepted effective prevention or treatment is available for preterm IVH, although recent studies have shown promising results. A previous clinical study showed that postnatal insulin-like growth factor-1 replacement might be a potential treatment for reducing comorbidities of prematurity (Ley et al., 2019). Following that study, Horsch et al. performed further *post-hoc* analyses to evaluate the effect of the replacement therapy on the incidence of brain injury in extremely preterm infants. They found that the potential protective effect of insulin-like growth factor-1 replacement on the occurrence of IVH was most pronounced in infants with no evidence of IVH at the start of treatment. To investigate the mechanisms and therapeutic strategies for IVH, Jinnai et al. established an intracranial collagenase injection model in P5 rats, which developed moderate brain injury affecting both the gray and white matter. These animals developed hyperactivity and showed reduced anxiety in the juvenile stage, which are relevant observations for mimicking IVH in preterm human infants. Post-hemorrhagic ventricular dilatation is a significant cause of death and disability, but few therapeutic options have so far been tested and new therapies are urgently needed for these infants. Mahoney et al. carried out a meta-analysis of 10 trials and concluded that drainage irrigation and fibrinolytic therapy appear to be the most likely candidates for improving outcomes after IVH.

FGR is a common complication of pregnancy often associated with neurological impairments, and there are currently no treatments for FGR. Kitase et al. established a novel FGR rat model that gradually restricted uterine/placental blood flow, which resulted in a 20% reduction in body weight among the offspring. They further investigated the potential of stem cell therapy for reversing the neurological impairment induced by FGR and found that intravenous administration of umbilical cord-derived mesenchymal stromal cells led to a significant amelioration of the reduced number of neurons and impaired behaviors induced by FGR. Glucocorticoids have been used prenatally to prevent complications in preterm infants, and Sutherland et al. investigated whether antenatal betamethasone altered brain development in a sheep model of FGR. They found that betamethasone administration resulted in independent subtle adverse effects on white matter development, which indicated that antenatal glucocorticoids should be administered with caution. The impact of toxic chemical exposure during the perinatal period on brain development has also attracted much attention. Newville et al. report that perinatal opioid (methadone) exposure exaggerated the peripheral immune responses and exacerbated inflammatory signaling, which in part contributed to neurological injury. Treatments to reduce inflammation could rescue the poor neural outcomes as a result of perinatal opioid exposure. Prenatal alcohol exposure is associated with different physical, behavioral, cognitive, and neurological impairments, and Almeida et al. summarize the current animal models of fetal alcohol spectrum disorders, including phenotypic features and neurodevelopmental deficits as well as the methodologies used to evaluate behavioral and anatomical alterations produced by prenatal alcohol exposure in rodents.

Perinatal brain injury-related neurological sequelae studies are also included in this collection. Wang et al. report that brainstem auditory evoked potentials at the age of 6 months can predict developmental delays in cognitive and motor skills, and thus they propose that the assessment of brainstem auditory evoked potentials may be used as a potential indicator for neurodevelopment. Xie et al. carried out a meta-analysis of expression datasets and identified a set of preeclampsia-driven molecular triggers that shift the developing brain toward a risk of autism spectrum disorders, and Yang et al. evaluated the effectiveness and safety of a clonidine adhesive patch for children with tic disorders in a real-world setting. O'Dea et al. summarize non-neurological organ dysfunction, which not only has a negative effect on the long-term outcome of neonatal brain injury, but may also influence the efficacy of treatments in the acute phase. Further evidence-based research is needed in order to optimize the management of brain injury, prevent further organ dysfunction, and reduce neurodevelopmental impairment.

In summary, this Research Topic has gathered contributions from the groups working in the field of perinatal brain injury and neurological sequelae, with an emphasis on perinatal etiologies of neonatal brain injury. Insight into the mechanisms that underlie the onset and progression of neonatal brain injury might help to identify new avenues for therapeutic intervention. We firmly believe that this is a significant period for research on all aspects of perinatal brain injuries and better preventions and treatments for these injuries will be forthcoming in the very near future.

## Author Contributions

CZ, CC, and CT wrote the manuscript, acted as editors to this Research Topic, and selected the articles described therein. All authors contributed to the article and approved the submitted version.

## Funding

Research in the authors' laboratories was supported by the National Natural Science Foundation of China (31761133015, U1704281, CZ), the Swedish Research Council (2018-02667, CZ), Action Medical Research (GN2796, CT), and the Medical Research Council (MR/T014725, CT).

## Conflict of Interest

The authors declare that the research was conducted in the absence of any commercial or financial relationships that could be construed as a potential conflict of interest.

## Publisher's Note

All claims expressed in this article are solely those of the authors and do not necessarily represent those of their affiliated organizations, or those of the publisher, the editors and the reviewers. Any product that may be evaluated in this article, or claim that may be made by its manufacturer, is not guaranteed or endorsed by the publisher.
